# Cannabis use, oral dysbiosis, and neurological disorders

**DOI:** 10.1515/nipt-2024-0012

**Published:** 2024-08-09

**Authors:** Amber A. Hazzard, Marice McCrorey, Tabinda Salman, Douglas E. Johnson, Zhenwu Luo, Xiaoyu Fu, Andrew P. Keegan, Andreana Benitez, Sylvia Fitting, Wei Jiang

**Affiliations:** Department of Microbiology and Immunology, 2345 Medical University of South Carolina, Charleston, SC, USA; Ralph H. Johnson VA Medical Center, Charleston, SC, USA; Department of Molecular and Cellular Biology and Pathobiology, 2345 Medical University of South Carolina, Charleston, SC, USA; Division of Multiple Sclerosis and Neuroimmunology, Department of Neurology, 2345 Medical University of South Carolina, Charleston, SC, USA; Department of Neurology, 2345 Medical University of South Carolina, Charleston, SC, USA; Department of Psychology & Neuroscience, University of North Carolina at Chapel Hill, Chapel Hill, NC, USA; Divison of Infectious Diseases, Department of Medicine, 2345 Medical University of South Carolina, Charleston, SC, USA

**Keywords:** cannabis, microbiome, dysbiosis, central nervous system, mental health

## Abstract

Cannabis (marijuana) is a leafy plant that has medical, recreational, and other uses. Cannabis is socially accepted and widely used throughout the United States. Though cannabis use is increasingly gaining popularity, studies detail the deleterious effects of chronic cannabis smoking on mental health, as well as the immunosuppressive properties of cannabinoids. Additionally, oral dysbiosis induced by cannabis smoking serves as a novel catalyst for neurological abnormalities, potentially possible through microbial translocation via the oral-brain axis. This review summarizes the effects and link of smoking cannabis on neurological abnormalities, immunity, and oral microbiome.

## Introduction

*Cannabis sativa* (cannabis), commonly known as marijuana, is a leafy flowering plant that has long been cultivated in Central Asia [1]. Cannabis was utilized by Central Asians for dietary needs, such as a source of food, fiber, and oil, as well as for religious, medicinal, and recreational purposes [2]. Present-day, cannabis is commonly used medicinally and recreationally. Notably, cannabis use disorder (CUD) is also common. CUD is characterized by symptoms including excessive cannabis consumption, unsuccessful attempts to cut down or control use, continued use despite social or interpersonal issues, and tolerance or withdrawal symptoms [3]. The CUD rate in the United States more than doubled from 4.1 % between 2001 and 2002 to 9.5 % between 2012 and 2013 [4]. This stark increase in CUD coincides with more lenient cannabis legislation. In 2012, citizens of Colorado and Washington State voted to legalize the recreational use of cannabis. Today, 24 states and the District of Columbia have fully legalized cannabis, while many others have approved medicinal cannabis use and/or decriminalized possession of cannabis in small amounts [5]. This continued shift toward fully legalizing cannabis throughout the United States is leading to an increased prevalence of cannabis users [6]. With increased legalization, there is more opportunity for chronic cannabis abuse, especially when users are exposed to cannabis in their adolescence [7]. It is well-documented that acute cannabis use causes changes in neural activity and psychological behaviors [8–10]. Although the effects of cannabis use have been widely studied and reported, there are still conflicting views in the literature regarding chronic cannabis exposure. The beneficial and detrimental effects are highly debated throughout the medical and scientific community. In this review, we summarized current studies related to cannabis smoking, immunity, oral microbiome, and central nervous system (CNS) abnormalities.

## Tetrahydrocannabinol (THC) is the primary psychoactive component in cannabis

Cannabis contains over 500 compounds, with over 60 being psychoactive cannabinoids [11]. Although many cannabinoids have now been identified, there are two main chemical constituents: tetrahydrocannabinol (THC) and cannabidiol (CBD). THC is the primary psychoactive component of cannabis, while CBD is the primary non-psychoactive component [12]. Evidence confirms that THC influences both immune and neural responses [13]. The effects CBD has on the CNS, however, are more elusive. CBD has well-known anti-inflammatory and neuroprotective properties [14]. In fact, a drug comprised of CBD is now an FDA-approved treatment for two rare forms of epilepsy [15]. Alternatively, CBD can increase cytokine production under certain conditions and cause lipopolysaccharide (LPS)-induced pulmonary inflammation *in vivo* [16, 17]. In humans, side effects of CBD use include liver and male reproductive dysfunction [18]. Further research is necessary to fully understand the immunological effects of CBD. For this review, we will focus on the immunologic effects of THC.

THC exerts its effects by interacting with specific endocannabinoid system (ECS) endogenous cannabinoid (CB) receptors, CB1 and CB2. The ECS plays a central role in CNS development, synaptic plasticity, and both endogenous and exogenous challenges to the neuromodulatory system [19]. These proteins have been purified and shown to be G-protein-coupled 7-transmembrane receptors (GPCRs), which typically modulate neuronal activity by affecting second messengers (e.g., adenylate cyclase) [20, 21]. The CB1 receptor is considered to be the most abundant GPCR in the brain; the high distribution of the CB1 receptor in neurons accounts for the majority of cannabis-associated behavioral actions [22]. CB1 receptor signaling activity on neuronal membranes contributes to a fine-tuned control of synaptic efficacy and plasticity [23]. Meanwhile, the CB2 receptor is found predominantly in cells in the immune system. CB2 is most prevalent in macrophages, though it is also found at lower levels in the CNS, with abundant expression in microglial cells and astrocytes [24–26]. Microglia have known involvement in the maturation of the brain’s neocortex region, which plays a role in learning and memory [27]. In mice, prenatal THC exposure (PTE) caused alterations in microglia function that lasted into young adulthood, which affected the overall development of the neocortex in young adults. In addition, PTE was found to have long-lasting effects on the overall brain, olfactory bulb, and diencephalon volume [28]. Studies have shown that THC inhibits CB1/CB2 second messengers cyclic-adenosine monophosphate (cAMP) in a reversible manner, as well as adenylate cyclase activity via GPCR activity both *in vitro* and in mice [29, 30]. These findings indicate potential neuronal signaling pathways that are affected by THC exposure and warrant further research into THC effects on downstream signaling of cAMP and adenylate cyclase activity.

Studies suggest THC may also be involved with ion transport through interactions with transient receptor potential (TRP) channels [31]. TRPs consist of transmembrane proteins that respond to various chemical and physical stimuli [32]. TRPs are implicated in physiologic and pathophysiologic conditions in the CNS and have been connected to neurodegenerative diseases such as Alzheimer’s disease and Parkinson’s disease [33]. THC was shown to be a functional agonist at the following TRP subsets in rats: TRPV2, TRPV3, TRPV4, and TRPM8 [34]. THC was not shown to influence TRPV1, however, which has analgesic effects [35]. THC interacting with TRPV2, TRPV3, TRPV4, and TRPM8 may contribute to the adverse effects of cannabis.

## Chronic cannabis smoking negatively impacts mental health

The adverse effects of cannabis have been summarized in several reviews [36–40]. Cannabis users have reported biphasic psychological effects of THC: they may involve either euphoria and relaxation or dysphoria and anxiety, the outcome of which principally depends on the dose level [41, 42]. Frequent users showed blunted psychotomimetic effects, perceptual alteration, cognitive impairment, anxiogenesis, and cortisol increase [8]. When challenged with acute psychosocial stress, lower doses of THC were associated with euphoria and relaxation while higher dosages correlated with increased negative psychological responses [43]. Additional adverse effects of chronic cannabis use include negative impacts on mental health and promotion of psychosis.

Routine cannabis use is associated with an increased risk of anxiety and depression [44], though causality has not been established. Cannabis exacerbates the development of schizophrenia, especially among people who have a genetic vulnerability, and is associated with other psychoses [45]. It has also been previously reported that the inhalation of THC increases striatal dopamine, which is thought to be responsible for psychotic symptoms [46, 47]. Furthermore, high-frequency and high-potency cannabis are independent factors that lead to a significantly higher risk of psychosis [48, 49]. Intravenous administration of THC may result in psychotic symptoms in a dose-dependent manner [41]. Likewise, the age of onset of cannabis usage may also impact the occurrence of psychosis. Early onset of psychosis was found to be potentially age-dependent, where cannabis abuse before the age of 15 correlated with a higher risk of experiencing an early psychotic episode [50]. There is a strong association between cannabis use and the risk of psychosis for those who start using cannabis in early adolescence. This may be because the brain developmental processes at this stage increases sensitivity to cannabis [48, 51]. Although studies suggest some populations benefit from using cannabis [52, 53], the negative short and long-term effects of cannabis use on mental health cannot be ignored. Moreover, the risks of cannabis use are related to the extent of usage, drug potency, age of exposure, and several other factors [54].

Furthermore, serotonin receptor 1b (5-HTR1B) expression was found to be upregulated with short-term usage of cannabis, but long-term cannabis abuse resulted in significant 5-HTR1B downregulation and behavioral changes [55]. These findings further indicate the frequency of cannabis use as a risk factor in the development of psychoses symptoms. In addition, polymorphisms in 5-HTR1B were found to be associated with the risk of schizophrenia development [56], indicating that cannabis use may also exacerbate psychosis symptoms in predisposed individuals. Further studies into the potential gene regulatory effects of cannabis are warranted.

## Cannabinoids are immunosuppressive

At the cellular level, cannabis exhibits immunosuppressive activity on several different immune cell types. Cannabinoids inhibited cytotoxic T lymphocytes (CTLs) by suppressing the cytotoxic activity [57] and lymphocyte maturation and differentiation [58]. Phytocannabinoids inhibited monocyte migration in isolated cells from cannabis users and expressed CB1 expression in monocytes [59]. Furthermore, cannabinoid receptor activation selectively inhibited the release of angiogenic factors from human lung macrophages [60], which could be explained by their reduced migratory function. Treatment of murine peritoneal macrophages with cannabis extracts has been shown to lead to impaired oxidative burst in response to LPS, which is characterized by down-regulated nitric oxide production and reduced levels of COX-2 and IL-1β [61]. The reduction of COX-2, cytokine production, and phagocytosis may account for impaired antimicrobial activity in alveolar macrophages from marijuana smokers in response to *Staphylococcus aureus* [62]. In this review, we summarize and discuss the immunosuppressive effects of THC.

THC can decrease the number of splenic dendritic cells (DCs), and alter the function of DCs by inhibiting MHC-II expression [63]. Consistently, THC is immunosuppressive and impairs host immune response to bacterial and viral infections. THC significantly inhibits natural killer (NK) cytotoxic activity, mediated by the CB1 and CB2 receptors [64]. Suppression of NK function was dependent on the concentration and duration of THC treatment [57, 65]. THC has also been shown to inhibit proliferation and induce apoptosis of other lymphocyte cell populations. Mice that received THC had significantly reduced proliferation of splenocytes following *in vitro* analysis of stimulating cells with anti-CD3 monoclonal antibody (mAb), Concanavalin A (ConA), and LPS *in vitro* [66]. In the same study, thymocytes, naive and activated splenocytes exposed to 10 mM or 20 mM of THC had significantly increased apoptosis in a dose-dependent manner [66]. Furthermore, THC decreased Bcl-2 and increased caspase-1 activity in naive and LPS-activated macrophages isolated from mouse splenocytes [67, 68]. Because THC and other cannabinoids induce apoptosis, inhibit cell proliferation, and suppress cytokine production, they are identified as anti-inflammatory molecules [69]. It is important to note, however, that patients with highly dysfunctional inflammatory activation (e.g., multiple sclerosis) may fail to exhibit the immunosuppressive effects of cannabis [70]. Outside of these conditions, the immunosuppressive effects of cannabis can decrease the robustness of the immune system.

The immunosuppressive effects of cannabinoids may lead to compromised immunologic competence in the respiratory system of cannabis smokers. This is indicated by an increased rate of respiratory infections and pneumonia [71, 72] and increased susceptibility to infection and poor outcomes of COVID-19 [73]. Cannabinoid-altered immunity depends on the duration of use; within the injured tissues, monocyte inflammatory responses were inhibited more extensively in individuals with chronic exposure to cannabis compared to short-term users or non-users [59]. Previous studies have demonstrated that THC treatment shifts the protective Th1 response to a non-protective Th2 response [74–76]. For example, *Legionella pneumophila* infection of mice induced IL-1, IL-12, and IFN-γ pro-inflammatory cytokines and Th1 immune response, whereas THC treatment prior to the infection suppressed immunity and early-stage IFN-γ, IL-12, and IL-12 receptor β2 responses during *L*. *pneumophila* infection [77]. More research is required to determine if the immunological suppression associated with chronic cannabis exposure can be reversed with cannabis extinction. Ultimately, the outcome of cannabis-induced inhibition of myeloid cell function may be an enhanced susceptibility to infectious disease and cancer in cannabis users.

### The cytokine response to THC may contribute to immunosuppression

Cytokines can be a double-edged sword that promote antimicrobial defenses against infections while accelerating pathogenesis [78]. In response to THC, macrophages altered the cytokine network, leading to a shift in the Th1/Th2 cytokine profile [79]. The anti-inflammatory effect of THC and other cannabinoids suggests that cannabinoids might be useful in mitigating the symptoms of autoimmunity and chronic inflammatory diseases [80, 81]. However, some studies have shown contradictory results. For example, one study showed that individuals with cannabis use disorder had an impaired oxidative balance and elevated levels of pro-inflammatory cytokine, including IL-1β, IL-6, IL-8, and TNF-α [82]. Therefore, the dosage, duration, and components (THC vs. CBD) of cannabis usage may lead to varied thresholds in response to cannabinoids, which may explain some of the contradictory results of cannabinoid-altered immune response [83]. Unfortunately, it is difficult to clinically determine the extent or longevity of the immunosuppression induced by cannabis and its consequences. This challenge is primarily due to multiple confounders, such as cannabis users are likely to be multiple drug users (e.g., tobacco smoking, alcohol abuse). The effects of cannabis use on cytokine production and concurrent immune response warrant further studies.

## Smoking cannabinoids induces oral dysbiosis

**Figure 1:**
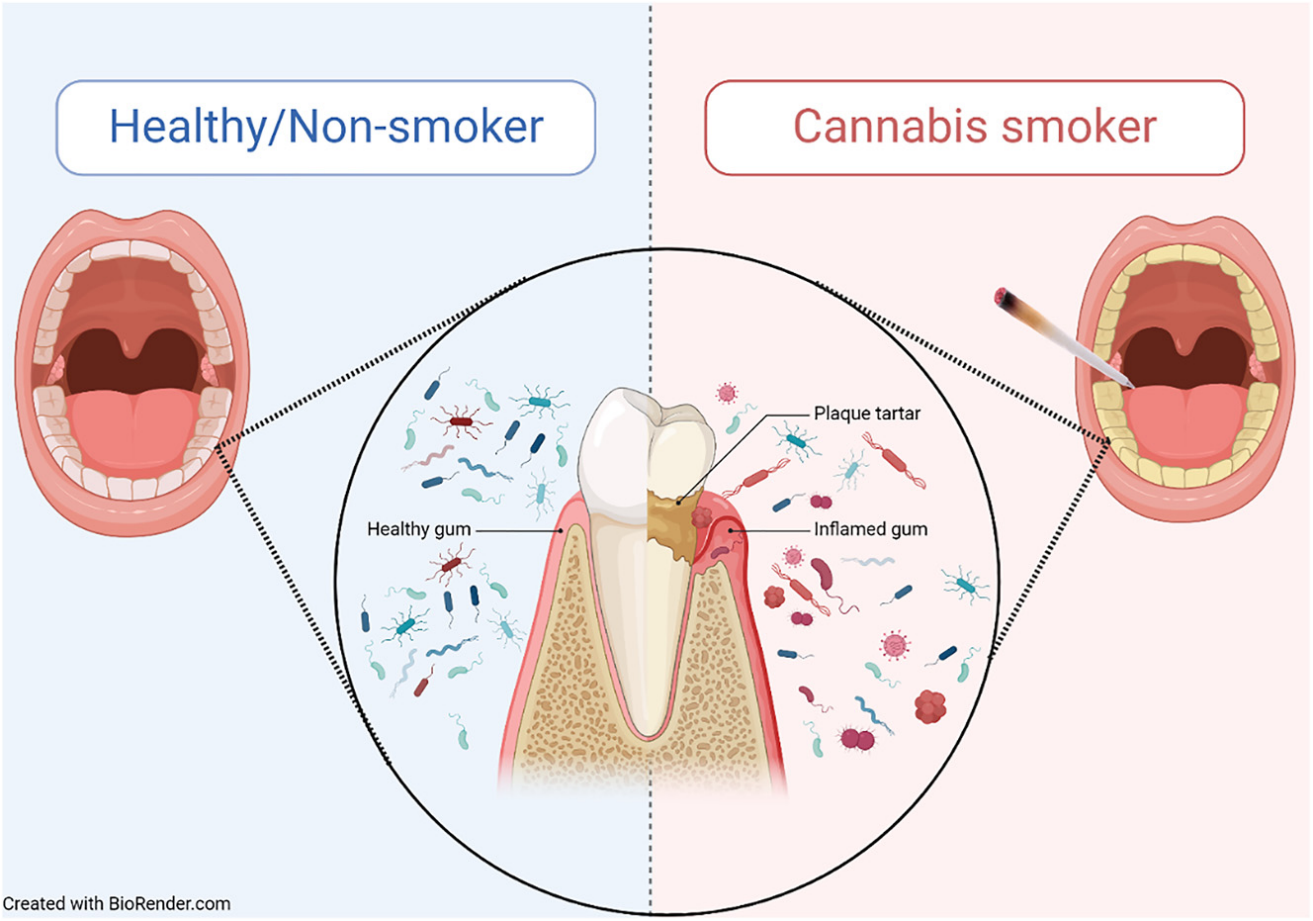
Smoking cannabis can induce oral dysbiosis.

The oral microbiome is one of the many microbiomes in the human body and can influence health and disease. It is made up of expansive populations of bacteria, viruses, fungi, and other microbes that colonize surfaces in the oral cavity, including the gingiva, teeth, cheeks, and tongue [84]. Under homeostatic conditions, microbes compete for resources [85], and this competition limits the growth of opportunistic and pathogenic microbes, lowering the chances of infection and encouraging a mutualistic relationship with healthy hosts [86]. Additional protection is provided by the oral epithelium, such as the gingiva, which serves as a selectively permeable membrane that prevents microbes and their metabolites from entering the bloodstream [87]. However, external factors, such as smoking, can cause a deviation from the typical resident oral microbes. This shift, known as oral dysbiosis, leads to increased pathogenic bacteria, biofilm formation, and host systemic responses [88]. Overall, increased pathogenic bacteria and worsened systemic host response can damage the epithelium, thus allowing microbes and their metabolites to enter systemic circulation. Smoking cannabis causes deviations from homeostasis that negatively impact the oral environment (Figure 1).

Smoking is the primary method for using cannabis [89]. Here, the smoking category does not include vaping. While vaping is becoming increasingly common, there is not yet enough data to comment on how the oral microbiome is affected by cannabis vaping exclusively. It is known, however, that smoking cannabis can change the oral environment. Cannabis users tend to show compromised oral health, with increased incidences of dental caries and periodontal diseases, increased rates of leukoedema, and increased prevalence and density of *Candida albicans* [82, 90]. Cannabis smoking may also act as a carcinogen. Specifically, cannabis smokers developed premalignant lesions in the oral mucosa with significantly increased decay on the surface of the teeth when compared to a non-cannabis-exposed control group [91]. A previous study of 903 participants from Dunedin and New Zealand found that cannabis smoking may be a risk factor for periodontal disease that is independent of the use of tobacco [92]. Oral bacteria have been linked to cardiovascular diseases, pre-term birth, and Alzheimer’s disease [93–95]. Therefore, the effects of cannabis on the oral environment can have a systemic impact. Based on these considerations, the oral microbiome that develops with cannabis use may modulate brain function directly through bacteria or product translocation into the brain or indirectly through pathways yet to be discovered.

### Pathogens may use the oral-brain axis to access the CNS

The oral microbiome has the second most abundant bacterial population after the intestines. The Human Oral Microbiome Database (HOMD) currently has 774 oral bacterial species cataloged, with 58 % being officially named, 16 % cultivated but not yet named, and 26 % uncultivated [96]. These bacterial genomes are categorized into 18 phyla; Absconditabacteria (SR1), Actinobacteria, Bacteriodetes, Chlamydiae, Chlorobi, Chloroflexi, Euyarchaeota, Firmicutes, Fusobacteria, Grancilibacteria (GN02), Ignavibacteriae, Lentisphaerae, Proteobacteria, Saccharibacteria (TM7), Spirochetes, Synergistetes, Tenericutes, and WPS-2 [96]. While the oral microbiota themselves have been well characterized, more research into their influence on the body is needed.

Several studies have confirmed the existence of the gut-brain axis: a bidirectional communication pathway between the microbes of the gastrointestinal (GI) tract and the brain. Though the GI tract encompasses the mouth, esophagus, stomach, intestines, and anus, most current literature focuses on intestinal microbiota. This focus is likely because the intestines having the largest microbial population at 10^12^ cells per milliliter of intestinal constituents [97]. Gut dysbiosis has been connected to various CNS abnormalities, including migraines, depression, autism, schizophrenia, and Alzheimer’s disease [98, 99]. Based on these findings, it is reasonable to postulate that the microbiota of other body cavities in the GI tract can also influence CNS abnormalities. Emerging evidence supports the existence of an oral-brain axis, similar to the gut-brain axis, that bacteria and/or their products can utilize to impact the CNS.

Cannabis use may influence the ability of pathogens to access the oral-brain axis. As addressed above, a decrease in host resistance may be the consequence of the immunosuppressive action of cannabinoids on the functionality of macrophages, T lymphocytes, and NK cells. Little is known concerning the potential of cannabinoids other than THC and CBD to alter immune functionality. The documented evidence that THC decreases salvation via CB1 activation and thus alters the innate anti-microbial activity of saliva *in vivo* indicates that cannabis use presents a potential risk of decreased resistance to infections in humans [100]. Studies suggest that marijuana is a co-factor that can increase the severity of infection by microbial agents by altering the host resistance [101]. Therefore, the effects of cannabis use on increased pathogen susceptibility in hosts pose an additional increased risk to immunocompromised individuals such as HIV-positive individuals [102]. Further studies addressing the enhancement of disease in immunocompromised individuals are warranted.

Smoking cannabis may lead to cannabis stomatitis, which includes changes in the oral epithelium, leukoedema of the buccal mucosa, and hyperkeratosis [103, 104]. Furthermore, opportunistic infectious bacteria from the mouth or gut may escape to new colonization sites and modulate the local environment. For example, *Arthrobacter* spp. and *Massilia timonae* have been isolated in patients from blood, cerebrospinal fluid, and bone [105, 106]. In periodontal bacterial overgrowth, increased presence of opportunistic bacterial infections and decreased inflammatory signals are observed in oral epithelial cells [107] exposed to E-cigarette aerosols. This infers that an altered local environment may stimulate a shift in the bacterial response. Many studies have demonstrated that the microbiome, microbiota-derived products, and related factors are correlated with or modulate neuro-psychiatric and behavioral disorders [108–113]. Among these, *Actinomyces meyeri* has been shown to cause brain abscesses and other types of CNS infections [114, 115], indicating that this organism may contribute directly to CNS damage or neurological abnormalities.

**Figure 2:**
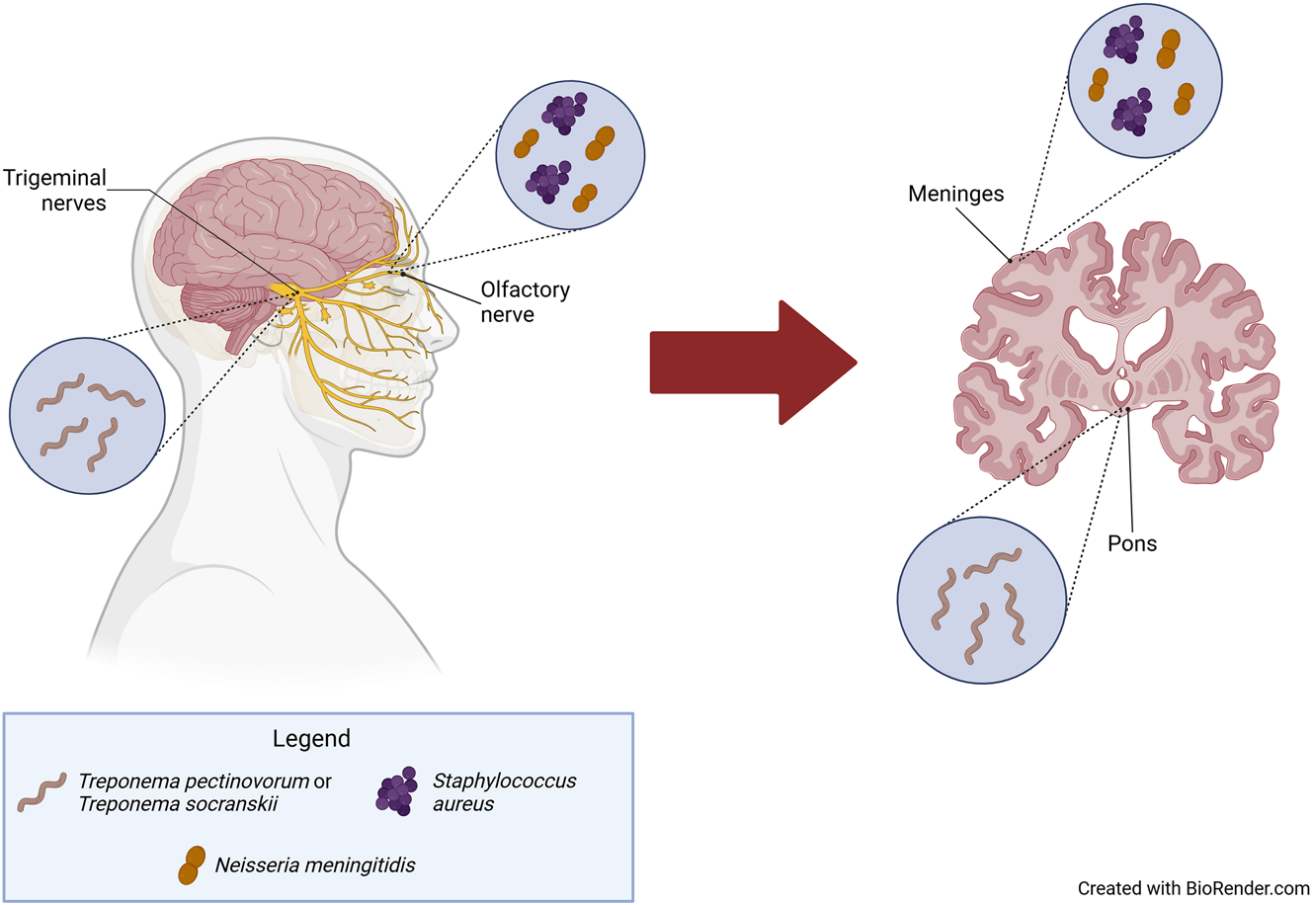
Bacteria translocation into the trigeminal and olfactory nerves of people with Alzheimer’s disease.

Many nerves lead from the oronasal cavity directly to the brain, including the trigeminal and olfactory nerves. These nerve pathways initiate in the nasal cavity at the olfactory neurepithelium and terminate at the central nervous system, which provides a direct route for pathogenic infection of the brain (Figure 2). Drugs can be directly transported from the nose to the brain along the olfactory and trigeminal nerve pathways to overcome the blood-brain barrier (BBB) [116]. The trigeminal nerve is thought to provide a route of entry for oral bacteria into the brain in Alzheimer’s disease. *Treponema pectinovorum* and/or *Treponema socranskii* are found in trigeminal ganglia and pons in some Alzheimer patients, indicating that oral *Treponema* may infect the brain via branches of the trigeminal nerve [117]. Another potential route is the olfactory nerve. *Neisseria meningitidis* can pass directly from the nasopharynx to the meninges through the olfactory nerve system [118]. In Alzheimer’s disease, hyposmia or anosmia are considered heralding symptoms [119, 120]. Olfactory ensheathing cells have many capabilities of macrophages to provide bactericidal protection against invasion via the oronasal route. They can migrate and engulf bacteria and can be activated to express inducible nitric oxide synthase in response to bacterial infiltration [121]. However, in some cases, compromised olfactory ensheathing cells provide a vehicle for bacterial transport. For example, *S*. *aureus* is able to penetrate the immunological defense of the damaged olfactory mucosa and infiltrate into the olfactory bulb [122].

### Microbial dysbiosis negatively impacts mental health

Previous studies suggest that LPS, produced by Gram-negative bacteria, can disrupt the BBB. The BBB cells respond to bacterial products (e.g., LPS) via Toll-like receptors (TLRs) (e.g., TLR2, TLR3, TLR4, and TLR6) expressed on the membranes of the constituent cells or intracellular expressed cells. LPS binding to TLR4 has been shown to increase the permeability of leukemia inhibitor factor from the blood to the brain [123]. Furthermore, activation of TLR2/6 leads to downregulation of the expression of tight junction proteins, such as occluding and claudin-5, on the cell membrane, leading to an increased permeability of the BBB [124]. Peripheral cytokine signals are amplified in the CNS by local inflammatory networks, which include inflammatory signal transduction pathways and induction of local cytokine production. In the brain, vagal afferents have been shown to mediate sickness behavior in response to peripherally administered LPS and IL-1 [125]. Additionally, endothelial cells and perivascular macrophages respond to circulating cytokines to induce expression of COX-2 [126, 127], which leads to neuronal injury in the setting of excitotoxicity [128]. After stimulation, cytokines in the brain are primarily produced by microglia [129] but also can be produced by oligodendrocytes [130] and astrocytes [131]. However, after acute inflammatory stimulation, increased CNS cytokine levels may play a role in protecting the brain. At the same time, under chronic immune activation, microglia may provide a source of inflammatory mediators that influence the brain neurotransmitter system and neuronal integrity [132].

LPS can induce microglia activation *in vivo* and *in vitro*. Microglia enhance neuronal survival by releasing trophic and anti-inflammatory factors, regulating brain development by enforcing the programmed elimination of neural cells. Activated microglia can produce inducible NO synthase (iNOS), reactive oxygen and nitrogen species (ROS/RNS), and MCP-1/CCL2, a chemokine involved in attracting peripheral immune cells to the brain; once activated, microglia cause progressive tyrosine hydroxylase and dopamine neuron loss [133, 134] potentially leading to behavioral changes. LPS and typhoid vaccination have been shown to affect basal ganglia activity that regulates microglia activity at neuronal synapses [135, 136]. In animal studies, after acute immune activation induced by LPS, indoleamine 2,3-dioxygenase activity is significantly increased at 24 h and peaks at 48 h in response to LPS administration. 2,3-dioxygenase has been proposed to mediate comorbid depression in inflammatory disorders, in which 5-hydroxytryptamine (5-HT) and other monoamines are released in the hypothalamus to mediate fever and sickness behavior [137]. Acute administration of cytokines, such as IFN-γ, IL-1β, TNF-α, and IL-6, increases 5-HT release in several brain regions, which could be mediated by increased 5-HT activity in addition to the cytokine-induced changes in 5-HT metabolism [138, 139]. Administration of inflammatory cytokines acutely increases 5-HT turnover in brain regions such as the cortex and nucleus accumbens, and these changes occur in concert with the appearance of later, more persistent depressive-like behaviors. For these reasons, LPS is an important bacterial component that can impact mental health, especially during microbial dysbiosis.

While evidence has linked inflammatory cytokines to the development of neuropsychiatric symptoms such as anxiety and depression, cannabis is generally thought to have an anti-inflammatory effect. For this reason, the inflammatory cytokine-mediated effects of cannabis on the CNS have not been discussed.

## Conclusions

Cannabis is the most widely used illicit drug in the world, with both therapeutic and recreational effects. However, chronic cannabis smoking can also have detrimental consequences on oral health and brain function. In this review, we have discussed the current documented effects smoking cannabis has on the immune system and brain function and development, which warrants further research into the risk factors associated with cannabis smoking. We highlighted evidence on how smoking cannabis alters the oral microbiome, leading to dysbiosis and increased levels of potentially harmful bacteria. We have also explored the possible mechanisms by which oral dysbiosis can affect the central nervous system through the oral-brain axis and contribute to cognitive impairment and neurodegenerative diseases, such as Alzheimer’s disease. Although the negative psychological and developmental risks associated with routine cannabis use are documented, there remains a strong push by researchers and medical doctors for its controlled use to treat psychosocial and inflammatory conditions in patients. However, this oral microbiome-centered connection between the brain and immune system warrants further immunological studies into the development of and risks of neuronal diseases associated with cannabis smoking.

## Supplementary Material

Supplementary Material Details

Supplementary Material Details
